# Pupae of the mega-diverse rove beetle tribe Staphylinini (Coleoptera, Staphylinidae): their traits and systematic significance

**DOI:** 10.3897/zookeys.877.35715

**Published:** 2019-10-02

**Authors:** Bernard Staniec, Ewa Pietrykowska-Tudruj

**Affiliations:** 1 Department of Zoology, Maria Curie-Skłodowska University, Akademicka 19, 20-033 Lublin, Poland Maria Curie-Skłodowska University Lublin Poland

**Keywords:** entomology, morphology, pupae, rove beetles, staphylinids

## Abstract

This paper is the first comprehensive work on the pupae of Staphylinidae. It is the first-ever attempt to employ the morphological characters of these pupae in phylogenetic analysis. The study shows that the external structures of Staphylinini pupae could be a useful, alternative source for assessing the relationships of certain taxa within a tribe. The paper also includes an illustrated key to the identification of pupae at the subtribe and generic levels (*Abemus*, *Acylophorus*, *Astrapaeus*, *Atanygnathus*, *Bisnius*, *Creophilus*, *Emus*, *Erichsonius*, *Gabrius*, *Hesperus*, *Heterothops*, *Neobisnius*, *Ocypus*, *Ontholestes*, *Philonthus*, *Quedius*, *Quedionuchus*, *Rabigus*, *Staphylinus*, and *Tasgius*) of the tribe Staphylinini, found in Europe. Based on current knowledge of the morphology of pupal stages of Staphylinini species, eight morphological pupal types are presented: Acylophorus, Astrapaeus, Atanygnathus, Erichsonius, Heterothops, Philonthus, Quedius and Staphylinus. The paper also comments on pupal habitat, phenology and morphology in the context of antipredator and environmental adaptations.

## Introduction

Rove beetles (Staphylinidae) are the largest family of organisms and dominate all ground-based cryptic microhabitats in every habitable landscape of the globe. Among insects, hyper-diverse families like rove beetles are the most difficult to analyse phylogenetically. They display an evolutionary radiation that took place 150–200 million years ago, since the fossil record indicates a notable diversity and abundance of Staphylinidae from at least the Late Jurassic (Solodovnikov et al. 2013). The overall pattern of rove beetle evolution is not well understood, and the phylogenetic system of this family is incomplete. Staphylinini, one of the largest tribes of rove beetles is an exception, however. It has recently been the focus of several phylogenetic studies involving adult morphological data, larval morphological data, the integration of adult morphologies of extinct and extant taxa, DNA sequences, as well as the integration of DNA sequences with adult morphology (e.g., Chani-Posse et al. 2018). Here we attempt to contribute a new set of data to the phylogeny of Staphylinini, relating to their pupae.

The difficulties of collecting and identifying pupae are due to their cryptic biology, and the need to link their morphology with the respective adults explains why little is known about the pupae and, in particular, why they have not been used for phylogenetic purposes.

In comparison with larvae or imagines, the pupae of Staphylinini are far poorer in morphological characters of diagnostic significance. The identification of pupae to species level is based on a small number of morphological characters revealed by morphometric analysis: the size and proportions of various body parts, the structure of the last abdominal segment, the structure and number of cuticular processes (including their range of variability), body microstructure and spiracular structure. Since rove beetles usually pupate in or near the habitats of their adults and larvae, the pupal biotope also provides a useful clue to their identification. Ecological data of this kind are especially helpful when comparing closely related species living in different habitats.

Knowledge of pupal morphology is fragmentary and varies in detail, depending on the subfamily. To date, pupae of the following subfamilies have been described, at least partially: Oxytelinae (almost 30 species), Steninae (6 species), Aleocharinae (a few species, only 3 in detail) and Paederinae (10 species), as well as Omaliinae, Tachyporinae, Scydmaeninae and Pselaphinae (single species) (e.g., Mank 1923; Hinton 1941; Welch 1966; Weinreich 1968; Żurańska 1973; Staniec 1993a, b, 1997, 2001b; Smoleński 1995; Carlton and Watrous 2009; Staniec et al. 2009a, 2010; Jałoszyński 2012; Zagaja et al. 2014). The most comprehensive knowledge of pupae is available for the subfamily Staphylininae, specifically its tribe Staphylinini, where the pupae of 103 species from 27 genera in 9 subtribes are known, albeit mostly from the Holarctic (Table 1).

In view of the above, the idea arose to compile a summary of existing knowledge of Staphylinini pupae. This is the first such comprehensive review worldwide dealing with Staphylinidae pupae. The main body of the paper is an illustrated key to assist the identification of known pupae of European Staphylinini at the subtribal and generic level. We also attempt to shed light on the potential importance of pupal characters in constructing phylogenetic hypotheses. This is the first attempt at applying the morphological characters of pupae to phylogenetic analysis and testing the hitherto accepted systematics at the generic level.

**Table 1.** Known pupal stages of Staphylinini species. Symbols: #–species considered in phylogenetic analysis. State of knowledge on morphology: very good-detailed and well-illustrated, descriptions reliable for diagnostics and sufficient for phylogenetic analysis; good-detailed enough descriptions with sufficient illustrations, reliable for diagnostics but not fully reliable for phylogenetic analysis; fair-moderately informative descriptions, maybe with sketchy illustrations (sometimes without), can be used for diagnostics but not for phylogenetic analysis; poor-hardly informative descriptions, mostly without any illustrations or no description with sketchy illustration, can be ambiguous even for diagnostic purposes.

Species	State of knowledge	References
**Subtribe Acylophorina**		
#*Acylophorus wagenschieberi* Kies.	good	Staniec (2005a)
**Subtribe Amblyopinina**		
#*Heterothops praevius* Er.	very good	Pietrykowska-Tudruj and Staniec (2006c)
**Subtribe Cyrtoquediina**		
#*Astrapaeus ulmi* (Rossi)	very good	Paulian (1941), Pietrykowska-Tudruj et al. (2014b)
**Subtribe Erichsoniina**		
*Erichsonius alumnus* Frank	fair	Schmidt (1996)
#*E. cinerascens* (Grav.)	very good	Pietrykowska-Tudruj and Staniec (2006c)
*E. pusio* (Horn)	poor	Schmidt (1994b)
**Subtribe Philonthina**		
*Belonuchus rufipennis* (Fabr.)	good	Mank (1923), Silvestri (1945)
*Bisnius cephalotes* Grav.	poor	Xambeu (1907)
#*B. fimetarius* (Grav.)	very good	Pietrykowska-Tudruj and Staniec (2011)
*B. nitidulus* (Grav.)	very good	Staniec and Kitowski (2004)
*B. sordidus* Grav.	poor	Xambeu (1907)
*Cafius canescens* (Mäklin)	poor	James et al. (1971)
*C. lithocharinus* (LeConte)	poor	James et al. (1971)
*C. luteipennis* Horn	poor	James et al. (1971)
*C. seminitens* Horn	poor	James et al. (1971)
*Gabrius osseticus* (Kolenati)	good	Pietrykowska-Tudruj and Staniec (2011)
*G. astutus* (Er.)	very good	Pietrykowska-Tudruj and Staniec (2010)
*G. splendidulus* (Grav.)	very good	Pietrykowska-Tudruj and Staniec (2007)
#*G. appendiculatus* Sharp	very good	Pietrykowska-Tudruj et al. (2014a)
#*Hesperus rufipennis* (Grav.)	good	Staniec (2004a)
*Neobisnius sobrinus* (Er.)	fair	Schmidt (1994a)
#*N. villosulus* (Steph.)	very good	Pietrykowska-Tudruj and Staniec (2007)
*Philonthus albipes* (Grav.)	good	Staniec (2002)
*P. atratus* (Grav.)	good	Pietrykowska-Tudruj and Staniec (2011)
*P. carbonarius* (Grav.)	good	Pietrykowska-Tudruj and Staniec (2011)
*P. chopardi* Cameron	poor	Tawfik et al. (1976a)
*P. cognatus* Steph.	good	Szujecki (1965)
*P. corvinus* Er.	good	Staniec (2003a)
*P. cruentatus* Gmelin	fair	Hunter et al. (1989)
*P. cyanipennis* Fab.	poor	Mank (1923)
*P. debilis* (Grav.)	good	Pietrykowska-Tudruj and Staniec (2011)
#*P. decorus* (Grav.)	very good	Verhoeff (1918), Pietrykowska-Tudruj and Staniec (2011)
*P. flavolimbatus* Er.	fair	Hunter et al. (1989)
*P. fumarius* (Grav.)	good	Staniec and Pietrykowska-Tudruj (2008b)
*P. laminatus* Creutzer	poor	Xambeu (1907, 1910)
*P. lepidus* (Grav.)	good	Staniec and Kitowski (2004)
*P. longicornis* Steph.	fair	Mank (1923), Tawfik et al. (1976b)
*P. micans* (Grav.)	good	Staniec (2003a)
*P. monivagus* Heer	poor	Xambeu (1900, 1907)
*P. natalensis* Boheman	?	Prins (1984)
*P. nigrita* (Grav.)	very good	Staniec (2001a), Staniec and Pietrykowska-Tudruj (2008a)
*P. nitidus* (Fabr.)	poor	Verhoeff 1920 (1919)
*P. politus* (L.)	very good	Pietrykowska-Tudruj and Staniec (2010)
*P. punctus* (Grav.)	good	Staniec (2003a)
*P. quisquiliarius* (Gyll.)	good	Hafez (1939), Staniec (2001a)
*P. rectangulus* Sharp	very good	Staniec (2004b)
*P. rubripennis* Steph.	very good	Staniec and Pietrykowska-Tudruj (2007)
*P. sanamus* Tott.	fair	Byrne (1993)
*P. sanguinolentus* (Grav.)	poor	Xambeu (1907, 1910)
*P. sericans* Grav.	poor	Mank (1923)
*P. splendens* Fabr.	poor	Xambeu (1894–97, 1907)
*P. succicola* Thoms.	good	Staniec (1999b, 2004b)
*P. tenuicornis* Rey	good	Staniec and Pietrykowska (2005a)
*P. turbidus* Er.	fair	Tawfik et al. (1976c)
*P. umbratilis* (Grav.)	good	Staniec and Kitowski (2004)
*P. varians* Payk.	very good	Xambeu (1907), Staniec (2002)
#*Rabigus tenuis* (Fabr.)	very good	Staniec and Pietrykowska-Tudruj (2008c)
*Remus sericeus* Holme	fair	Paulian (1941)
**Subtribe Quediina**		
#*Quedionuchus plagiatus* Mann.	good	Saalas (1917), Staniec (1996)
*Quedius abietum* Kies.	poor	Xambeu (1900)
*Q. brevicornis* (Thom.)	good	Drugmand (1988), Staniec (2003b)
*Q. brevis* Er.	very good	Pietrykowska-Tudruj and Staniec (2006b)
*Q. capucinus* (Grav.)	poor	Voris (1939b)
*Q. cruentus* (Ol.)	good	Staniec and Pietrykowska (2005b)
*Q. curtipennis* Bernh.	good	Outerelo (1978)
#*Q. cinctus* (Payk.)	very good	Pietrykowska-Tudruj and Staniec (2010)
*Q. dilatatus* (Fabr.)	poor	Strassen (1957), Vorst and Heijerman (2015)
*Q. fulgidus* Fabricius	poor	Xambeu (1910)
*Q. fuliginosus* (Grav.)	good	Staniec (1999a)
#*Q. fumatus* (Steph.)	good	Staniec (1999a)
*Q. humeralis* Steph.	good	Staniec (1999a)
*Q. levicollis* Brullé	poor	Waterhouse (1836), Lesne (1890)
#*Q. microps* (Grav.)	very good	Pietrykowska-Tudruj and Staniec (2006b)
*Q. mesomelinus* (Marsh.)	good	Staniec (1999a)
*Q. molochinus* (Grav.)	poor	Voris (1939b)
*Q. ochripennis* Ménétriés	fair	Falcoz (1914), Xambeu (1899, 1910), Beier and Strouhal (1928)
*Q. umbrinus* Er.	poor	Mjöberg (1906)
*Q. scintillans Grav.*	poor	Perris (1853)
*Q. semiobscurus* Marsh.	poor	Xambeu (1910)
*Q. spelaeus spelaeus* Horn.	good	Moseley et al. (2006)
**Subtribe Staphylinina**		
#*Abemus chloropterus* (Panz.)	fair	Boháč (1982)
#*Creophilus maxillosus* (L.)	poor	Dajoz and Caussanel (1968), Voris (1939a), present study
*Emus hirtus* (L.)	poor	present study
*Hadropinus fossor* Sharp	fair	Shibata (1965)
*Ocypus aeneocephalus* (De Geer)	poor	Boháč (1982)
#*O. fulvipennis* (Er.)	good	Staniec et al. (2009b)
*O. fuscatus* (Grav.)	fair	Boháč (1982)
*O. italicus* (Arag.)	poor	Boháč (1987)
*O. nitens* Schrank	good	Verhoeff (1918), Boháč (1982), present study
*O. olens* (O. F. Müll.)	poor	Orth et al. (1976)
#*Ontholestes murinus* (L.)	good	Staniec (2004b)
*O. cingulatus* (Grav.)	poor	Voris (1939a)
#*Platydracus tomentosus* (Grav.)	fair	Voris (1939a), Schmidt (1994b)
*P. cinnamopterus* (Grav.)	?	Lesage (1977)
*P. comes* (LeConte)	poor	Voris (1939a)
*P. maculosus* (Grav.)	poor	Voris (1939a)
*P. viridanus* Horn	poor	Voris (1939a)
*Staphylinus caesareus* Ced.	fair	Boháč (1982)
#*S. erythropterus* L.	very good	Szujecki (1960), Boháč (1982), Pietrykowska-Tudruj and Staniec (2012)
#*Tasgius melanarius* (Herr)	very good	Staniec and Pietrykowska (2005b)
**Subtribe Tanygnathina**		
#*Atanygnathus terminalis* (Er.)	very good	Staniec (2005b)
**Subtribe Xanthopygina**		
*Smilax deneinephyto* Chatzimanolis	poor	Eidmann (1937)
*Triacrus dilatus* Nordm.	poor	Wasmann (1902)

## Material and methods

### Description of pupal morphology and key to subtribes and genera of the tribe Staphylinini

The diagnostic characters given in this paper were established generally on the basis of current knowledge of the pupal stage in Staphylinini. The key covers 8 subtribes and 20 genera (highlighted in Table 1 by an asterisk) of pupae found in Europe. Most of the data and all the drawings have been taken from papers by the present authors, published between 1996–2014. Some information relating to *Abemus* and in part to: *Creophilus*, *Ocypus*, *Philonthus*, *Platydracus*, *Quedius* and *Staphylinus*, has been taken from papers by other authors (e.g., Szujecki 1965; Dajoz and Caussanel 1968; Orth et al. 1976; Boháč 1982, 1987; Schmidt 1994b; Moseley et al. 2006; Vorst and Heijerman 2015). The paper also includes new information on the pupa of *Emus hirtus* (L.) which was hitherto unknown, and photographs and notes supplementing existing descriptions of the pupae of nine genera, represented by species: *Acylophorus wagenschieberi* Kies., *Creophilus maxillosus* (L.), *Gabrius appendiculatus* Sharp, *Ocypus fulvipennis* (Er.), *Quedius microps* (Grav.), *Rabigus tenuis* (Fabr.) and *Staphylinus erythropterus* L. The photographs were taken with an Olympus DP72 digital camera mounted to an Olympus SZX16 compound microscope (Fig. 7) or with a VEGA3 TESCAN SEM (Figs 2a, 2b, 6b, 13, 14), and corrected using CorelDRAW Graphics Suite X6.

Material that was here examined for the first time includes one pupa of *C. maxillosus* (male) and one exuvium of *E. hirtus*, obtained from the collection of the Zoological Museum of the University of Copenhagen, Denmark (**NHMD**, the Natural History Museum of Denmark). The pupae of these four species, as well as others previously described by the authors, are deposited in the collection of the Department of Zoology, Marie Curie Skłodowska University, Lublin, Poland.

### Terminology, measurements and abbreviations

**Figure 1–13.**
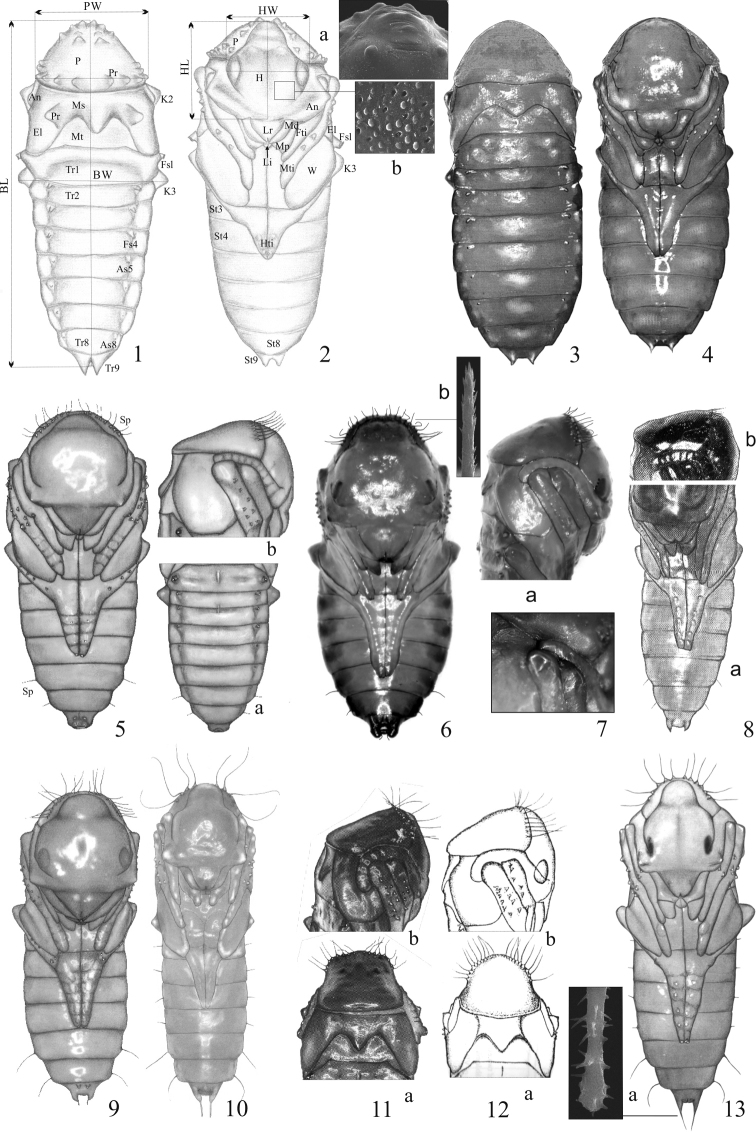
Pupae; dorsal aspect (**1, 3**), ventral aspect (**2, 4, 5, 6, 9, 10, 13**), lower part in dorsal aspect (**5a**), lower part in ventral aspect (**8a**), upper part in dorsal aspect (**11a, 12a**), upper part in lateral aspect (**5b, 6a, 11b, 12b**), cuticular projection (**2a, 6b, 13a**), microstructure of the head (**2b**) **1–2b***Acylophorus wagenschieberi***3, 4***Astrapaeus ulmi***5–5b***Ontholestes murinus***6–6b***Staphylinus erythropterus***7***Creophilus maxillosus*, tibiae I and II and antenna **8a, b***Ocypus fulvipennis***9***Tasgius melanarius***10***Neobisnius villosulus***11a, b***Philonthus decorus***12a, b***P. succicola***13, 13a***Rabigus tenuis*. Abbreviations: An antenna, As atrophied spiracle, BL body length, BW body width, El elytra, Fs functional spiracle, Fti fore tibia, H head, HL head length, Hti hind tibia, HW head width, K knee, Li labium, Lr labrum, Md mandible, Mp maxillary palp, Ms mesonotum, Mt metanotum, Mti mid tibia, P pronotum, Pr protuberance, PW pronotum width, Sp setiform projection, St sternite, Tr tergite, W wing.

The terminology follows Staniec (1999a, b, 2002) and Pietrykowska-Tudruj and Staniec (2011). Measurements and their abbreviations are after Pietrykowska-Tudruj and Staniec (2012) and Pietrykowska-Tudruj et al. (2014b) marked on Figures 1, 2, 5, 18, 26, and 32 as follows: **BL** body length, **BW** body width, **HW** head width, **HL** head length, **PW** pronotum width, Abbreviations of the body parts as follows: **A** accessory, **An** antenna, **As** atrophied spiracle, **El** elytra, **Fs** functional spiracle, **Fti** fore tibia, **H** head, **Hti** hind tibia, **K** knee, **Li** labium, **Lr** labrum, **Md** mandible, **Mp** maxillary palp, **Ms** mesonotum, **Mt** metanotum, **Mti** mid tibia, **P** pronotum, **Pr** protuberance, **Sap** spiracular appendage, **S** spine, **Sp** setiform projection, **St** sternite, **Tp** terminal prolongation, **Tr** tergite, **W** wing, **Vp** ventral prolongation.

### Phylogenetic analysis

The phylogenetic analysis was carried out at the generic level. A data matrix was assembled in Nexus Data Editor for Windows v. 0.5.0 (Page 2001) that included 29 morphological characters of the well-known pupae from 22 species as the ingroup belonging to 20 genera of Staphylinini from 8 subtribes (Table 4). Each genus is represented by one species, except for *Quedius* (three species from the subgenera *Distichalius*, *Raphirus*, *Microsaurus*). As the pupae of the species from these three subgenera differ in certain morphological characters, they have been included in the data matrix. Some genera (*Belonuchus*, *Cafius*, *Emus*, *Remus*, *Smilax*, *Triacrus*, *Hadropinus*) have not been included because the available morphological data on their pupae are too fragmentary and superficial, and therefore deemed unreliable. The pupa of *Hypnogyra angularis* (Ganglbauer, 1895) from the tribe Xantholinini is added as an outgroup to the tribe Staphylinini (Staniec and Pietrykowska 2005a, Pietrykowska-Tudruj and Staniec 2006a, unpublished data). Inapplicable characters are assigned a gap value (‘−’) and treated as equivalent to missing data (‘?’). The matrix was analysed in TNT (Goloboff et al. 2008) under settings as follows: the ‘traditional search’ option for the parsimony analysis – 1000 replicates with tree bisection reconnection (TBR) branch swapping and saving 1000 trees per replicate, zero-length branches collapsed, all characters were treated as unordered and equally weighted.

## Results

### Diagnostic description of pupae of the tribe Staphylinini

Pupa obtect. Body clearly slender, almost cylindrical and weakly sclerotised (e.g., *Neobisnius*), or moderately elongate, slender and moderately sclerotised (e.g., *Erichsonius*, *Gabrius*, *Heterothops*, some species of *Philonthus*), or moderately stocky and well sclerotised (e.g., *Astrapaeus*, *Quedius*) to extremely stocky and strongly sclerotised (e.g., *Atanygnathus*). Colour: almost white or pale yellow shortly after pupation; from dark yellow to reddish brown a few days after pupation; usually almost black just prior to emergence of imago.

Head directed ventrally towards thorax, without any setiform projection or spines, rarely with a few protuberances. Labrum usually V-shaped, exceptionally U-shaped, with short, longitudinal groove running from its anterior margin. Mandibles elongate, usually pointing posteriorly, falcate or almost straight. Maxillae usually moderately long. Antennae curved, rest on knees of fore and mid legs; apex usually protruding beyond knee of mid tibia. Scutiform pronotum widest at the base, usually about as wide as long with 6–32 setiform projections, or a pair of micro spines or 8–26 protuberances, sometimes with no structures. Mesonotum separated from pronotum by a furrow, distinctly wider than long. Metanotum narrower than mesonotum with deeply bisinuate anterior margin. Elytra shortened. Wings protruding to ventral side. Apex of wings protruding at most beyond posterior margin of I (morphologically III), clearly visible abdominal segment. Tibiae and tarsi directed obliquely towards body middle. All tibiae, or only some of them with pointed protuberances. Hind tarsi at most reaching midpoint of V (morphologically VII), clearly visible abdominal segment.

Abdomen with 9 somewhat flattened tergites and 7 convex sternites visible. Abdominal tergite I wider than others and about twice as long as tergite II. Abdominal shape of three kinds: arcuate, with parallel sides or funnel-shaped. Sides of abdomen with: spines on segments II–VIII or II–VII, or setiform projections on segments III–VIII or VII–VIII. Rarely abdomen without any lateral cuticular projections. Last segment usually strongly protruding into two terminal, elongated prolongations, sometimes weakly protruding into two triangular prolongations, exceptionally without prolongations. Terminal sternite with well-marked sexual dimorphism. Gonotheca in female double, in male single. In female pupae, terminal sternite often with a pair of prolongations. Abdominal tergites I–IV with tuberculate, functional spiracles, the first pair usually situated more laterally, most often larger and protruding farther than the others; tergites V–VIII with externally visible, but apparently atrophied spiracles.

### Comparison

The following crucial characters distinguish the pupae of the tribe Staphylinini from the tribe Xantholinini within the subfamily Staphylininae for which the pupae are known: abdominal segments divided laterally into ventral and dorsal sclerites (not grown into uniform rings); body with setiform projections, spines or protuberances, apart from the genus *Astrapaeus* which has no cuticular processes (Staniec and Pietrykowska 2005a, Pietrykowska-Tudruj and Staniec 2006a). The combination of characters distinguishing the pupae of Staphylinini within the family Staphylinidae, i.e., the subfamilies Aleocharinae, Omaliinae, Oxyporinae, Oxytelinae, Paederinae, Steninae and Tachyporinae for which the pupae are known, includes: i. exarate pupa; ii. no projections whatsoever on head; iii. short labium; iv. lack of short setae on dorsal and/or ventral part of abdominal sclerites; v. lack of setae on hind margin of prothorax.

### Morphological types of pupae of the tribe Staphylinini

Based on current knowledge of the pupal morphology of Staphylinini species, eight morphological types were distinguished: Acylophorus (genus: *Acylophorus*), *Astrapaeus* (species: *Astrapaeus ulmi* (Rossi)), Atanygnathus (species: *Atanygnathus terminalis* (Er.)), Erichsonius (genus: *Erichsonius*), Heterothops (genus: *Heterothops*), Philonthus (genera: *Bisnius*, *Gabrius*, *Hesperus*, *Neobisnius*, *Philonthus* and *Rabigus*), Quedius (genera: *Quedius* and *Quedionuchus*) and Staphylinus (genera: *Abemus*, *Creophilus*, *Emus*, *Ocypus*, *Ontholestes*, *Staphylinus* and *Tasgius*). These types take into consideration pupae from 20 genera, most of which have been described by the present authors. The diagnosis of the types is presented in Table 2.

**Table 2.** Characters of the morphological types of pupae of the tribe Staphylinini. Symbols and abbreviations: N number, MS moderately stocky, MSc moderately sclerotized, HS heavily stocky, SSc strongly sclerotised, WS well stocky, WSc well sclerotized, abs absent, pre present, S spine, Sp setiform projection, – no data, for abbreviations of the body parts see Material and methods.

Type of pupa	Body shape/cuticula	Cuticular processes (Cp)		Protuberance location	Segment IX: Vp ♀ (A)/Tp ♀♂ (A)	Special characteristic
		pronotum	abdomen			
		type: amount/ length/shape/A	type: amount/N of Sg with Cp/ length/shape/A			
Acylophorus	MS/SSc	abs	abs	H, P, Ms*	pre (-)/ pre (abs)	H rhomboidal, I^st^ pair of Fs distinctly bigger than the others
Astrapaeus	MS/WSc	abs	abs	Mti, Hti	abs/pre (pre)	Lr U-shaped
Atanygnathus	HS/SSc	abs	S: 12/II–VII/long/straight/pre	P, Ms**	abs/pre (abs)	Md rounded apically, Mp strongly elongate, Sap of Fs, I^st^ pair of Fs strongly protruding laterally
Erichsonius	MS/MSc	abs	S: 14/I–III/equal/straight/pre	P, Mti	pre (usually abs)/pre (usually abs)	–
Heterothops	MS/MSc	abs	S: 14/II–VIII/ equal/straight/ pre	H, P, Mti, Hti	pre (pre)/pre (pre)	H small, W short
Philonthus	diverse character	Sp: 6–24 /long/ usually decurved/-	Sp: 12/III–VIII or 4/VII–VIII/ short III–VI; long VII–VIII/ straight III–VI; curved VII–VIII /pre	Ft, Mti, Hti	pre (pre)/pre (pre)	–
Quedius	MS or WS/MSc or SSc	abs or pre S: 2/tiny•/straight/-	S: 14 II–VIII/ equal/straight/ smooth#.	Mti, Hti	pre (abs #)/pre (abs #)	–
Staphylinus	WS/WSc	Sp: 12–32/ short••/straight or slightly wavy/-	Sp: 4 VII–VIII/ short/straight or slightly decurved/-	–	all characteristic pre or abs	H relatively wide

*the number of protuberances 2, 26, 3, respectively, **the number of protuberances 7, about 26, 3, respectively, •45–144 μm, ••length less than half of pronotum; #exceptionally in *Q. plagiatus* with accessory

### Key to pupae of Staphylinini

**Key to subtribes of Staphylinini**

**Table:** 

1	Body without any spines or setiform projections	**2**
–	Pronotum or/and abdomen with spines or setiform projections	**3**
2	Pronotum, head and mesonotum with protuberances (Figs 1, 1A, 2). Abdomen tapering gradually from first to last (IX) segment (Fig. 1)	**Acylophorina**, genus: *Acylophorus* Nordmann, 1837
–	Pronotum without any protuberances (Figs 3, 4). Abdomen arcuate, widening gradually from segment I to V, then tapering to terminal segment	**Cyrtoquediina**, genus: *Astrapaeus* Gravenhorst, 1802
3	Pronotum with setiform projections located on protuberances at anterior margin (Fig. 28)	**4**
–	Pronotum without setiform projections, at most with a pair of tiny spines (S) (Figs 22, 23, 29) or globular protuberances (Figs 18–20)	**5**
4	Setiform projections on pronotum (Figs 5–9) straight or slightly wavy and short, shorter than half pronotum length. Abdominal segments VII–VIII, each with a pair of usually short, straight or slightly curved setiform projections (Sp) (Figs 5, 6, 8a)	** Staphylinina **
–	Setiform projections on pronotum (Figs 10–17) distinctly wavy and long, at least as long as half pronotum length, lateral projections usually distinctly decurved. Abdominal segments III–VIII or VII–VIII each bearing a pair of setiform projections or (exceptionally) spines	** Philonthina **
5	Abdominal segments II–VII each with a pair of spines on sides (Figs 18, 19). Functional spiracles of abdominal segments II–IV each with a unique appendage (Fig. 32)	**Tanygnathina**, genus: *Atanygnathus* Jakobson, 1909
–	Abdominal segments II–VIII each with a pair of spines on sides (Figs 22–24). Functional spiracles of abdominal segments II–IV without appendages	**6**
6	Pronotum without protuberances	** Quediina **
–	Pronotum with tiny, globular protuberances (Fig. 20)	**7**
7	Pronotum at most with 10 protuberances, head large, without protuberances (Fig. 21)	**Erichsoniina**, genus: *Erichsonius* Fauvel, 1874
–	Pronotum with at least 20 protuberances, head small, with 2 protuberances (Fig. 20)	**Amblyopinina**, genus: *Heterothops* Stephens, 1829

**Figure 14–24.**
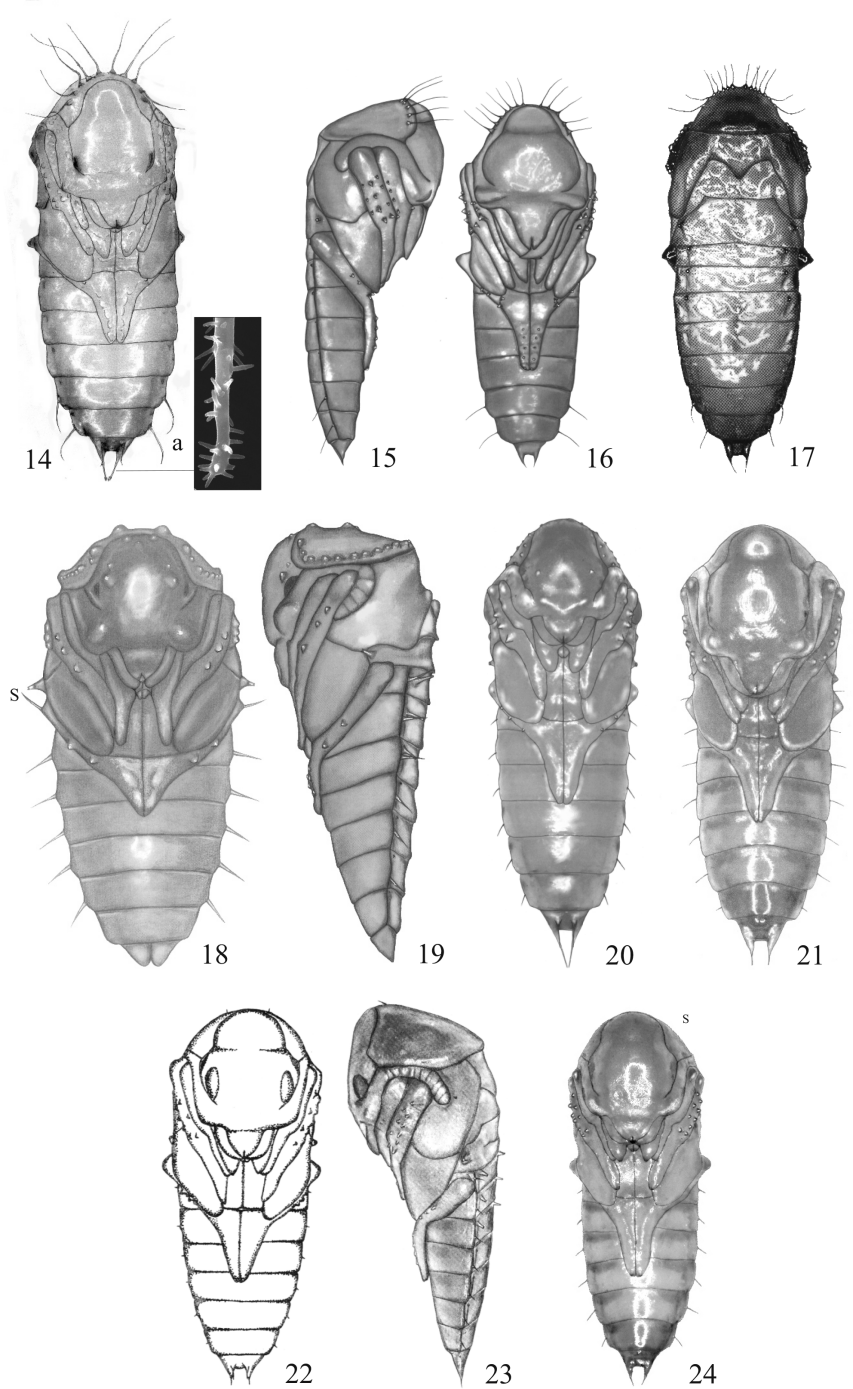
Pupae; dorsal aspect (**17**), lateral aspect (**15, 19, 23**), ventral aspect (**14, 16, 18, 20, 21, 22, 24**), accessories of terminal prolongation (**14a**) **14, 14a***Gabrius appendiculatus***15, 16***Hesperus rufipennis***17***Bisnius fimetarius***18, 19***Atanygnathus terminalis***20***Heterothops praevius***21***Erichsonius cinerascens***22***Quedius fumatus***23***Q. cinctus***24***Q. microps*.

### Key to genera of Staphylinini

**Subtribe Staphylinina**

**Table:** 

1	Abdominal segment IX without terminal prolongations (Fig. 25)	**2**
–	Abdominal segment IX with a pair of short terminal prolongations, each protruding into two recurved accessories (A) (Figs 26–27)	**4**
2	Pronotum with 13–16 setiform projections. Head relatively wide (Fig. 5). Antennae short, at most reaching apex of mid tibia (Fig. 5b). Abdomen tapering below segment IV (Fig. 5a). Terminal sternite of female as in Fig. 25. Pupal cocoon as in Fig. 33. BL: 8.63–9.25 mm; BW: 4.00–4.50 mm; HW: 2.70–3.00 mm; PW: 2.80–3.00 mm. Biotope: remains of large animals, excrement and decaying plant matter	***Ontholestes* Gangalbauer, 1895**
–	Pronotum with more than 16 setiform projections	**3**
3	Pronotum with 20 setiform projections. Antennae protruding slightly beyond apex of mid tibia. Abdomen tapering below segment IV. BL: 7.5 mm. Biotopes: old deciduous forests, rotting remains of deciduous trees, moss at the base of trees, leaf litter and decaying plant matter	***Abemus* Mulsant & Rey, 1876**
–	Pronotum with about 30 setiform projections; two specimens examined. Biotopes: open and wooded areas, excrement, decaying plant matter and carrion	***Emus* Leach, 1819**
4	Antennae reaching at most to apex of mid tibia (Fig. 7). Body relatively stocky. Pronotum with 22–32 setiform projections at the anterior margin. Hind legs reaching half way along abdominal sternite VI (well visible IV). BL: 11–15 mm. Biotopes: remains of large animals, excrement and decaying plant matter	***Creophilus* Leach, 1819**
–	Antennae protruding at least slightly beyond apex of mid tibia (Figs 6a, 8b). If antenna reaching at most to apex of mid tibia, then pronotum with under 20 setiform projections	***Ocypus* Leach, 1819, *Staphylinus* Linnaeus, 1758, *Tasgius* Stephens, 1829**

**Figure 25–34.**
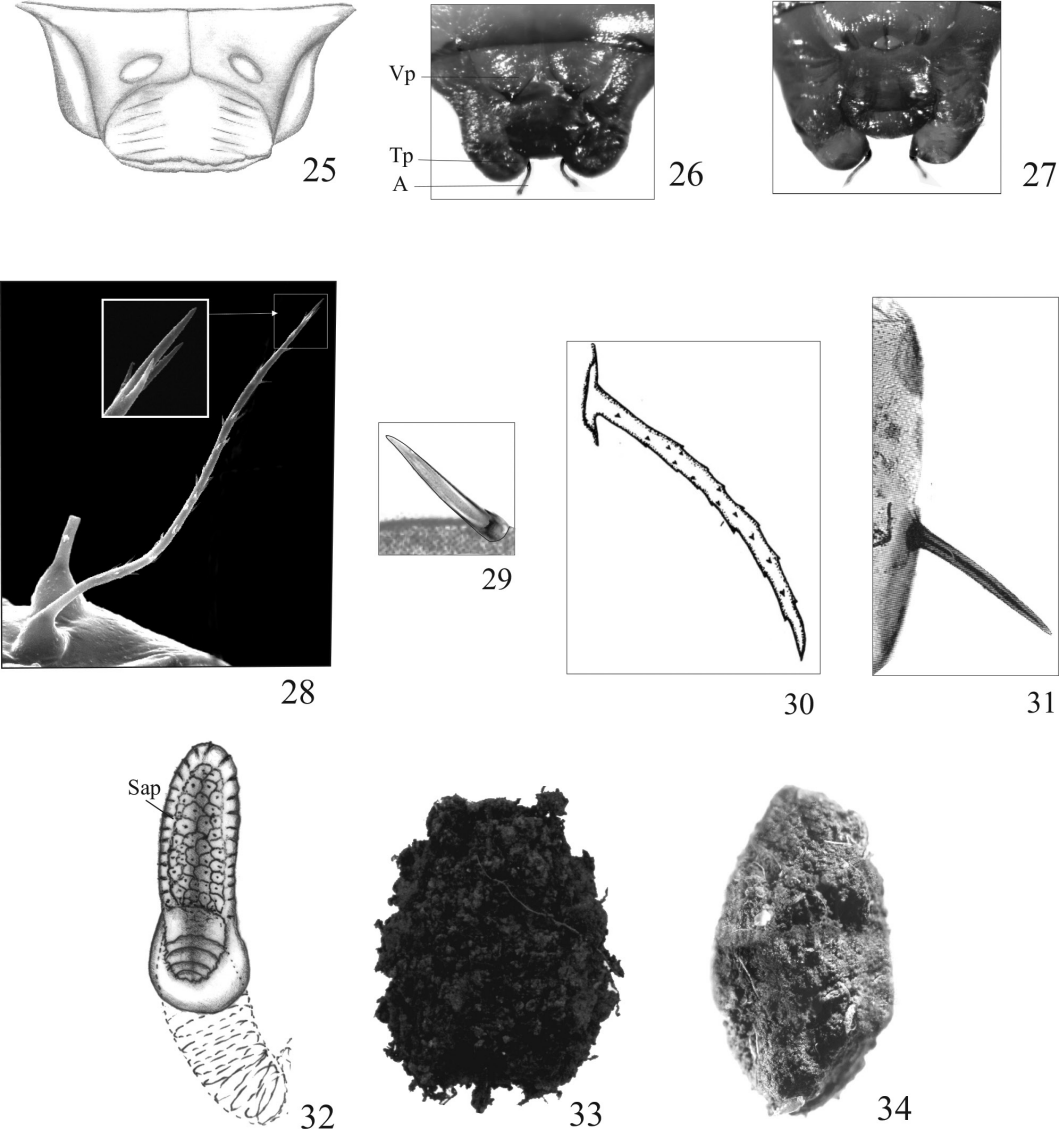
Pupae; terminal sternites (**25–27**), setiform projection of pronotum (**28**), spine of pronotum (**29**), spine of abdomen (**30, 31**), functional spiracle (**32**). Cocoon (**33, 34**) **25***Ontholestes murinus*, female **26, 27***Staphylinus erythropterus*, female (**26**), male (**27**) **28***Gabrius appendiculatus***29***Quedius cinctus***30***Quedionuchus plagiatus***31***Quedius cinctus***32***Atanygnathus terminalis*, III pair **33***Ontholestes murinus***34***Rabigus tenuis*. Abbreviations: A accessory, S spine, Sap spiracular appendage, Tp terminal prolongation, Vp ventral prolongation.

### Subtribe Philonthina

**Table:** 

1	Body slender, lateral margins almost parallel (Fig. 10). Colour light yellow, cuticula weakly sclerotised. Head 1.7 times as long as wide. Antennae half as long as elytra. Pronotum 1.5 times as broad as long, with long 7–8 setiform projections. Hind tarsi reaching posterior margin of abdominal sternite V (well visible III). BL: 2.76–3.22 mm; BW: 0.91–1.07 mm. Biotopes: usually moist river and stream banks, under plant debris, slime and stones	***Neobisnius* Gangalbauer, 1895**
–	Body moderately slender or stocky, abdomen tapering below abdominal segments III or V to terminal prolongations	**2**
2	Segments III–VI each with short spines (Fig. 13). Pupal cocoon present (Fig. 34). Antennae nearly two-thirds as long as elytra. Pronotum with 10–12 setiform projections. Mid tarsi protruding distinctly beyond posterior margin of abdominal sternite III (well visible I) (Fig. 13). Spines of segments III–VI smooth, at least 3 x shorter than segment. BL: 3.4–3.8 mm. BW: 1.51–1.72 mm; HW: 0.73–0.82 mm; BW: 0.90–1.00 mm. Biotopes: sunny, moist places, on clayey and loess soil, sparsely covered by grasses or devoid of any vegetation, in plant debris, under stones	***Rabigus* Mulsant & Rey, 1876**
–	Segments III–VI each with setiform projections (Fig. 13) or without any cuticular projections. Pupal cocoon only exceptionally present	**3**
3	Labrum elongated (Figs 15, 16). Mandibles in both sexes long, crossed in apical part. Antennae not reaching midpoint of elytra (Fig. 15); Pronotum with 10–15 setiform projections. Hind tarsi reaching midpoint of abdominal sternite VI (well visible IV) (Fig. 16). Abdomen relatively slender, tapering below segment V. Sternite IX in female without ventral prolongations; terminal abdominal prolongation (Tp) sharpened apically. BL: 6.75–7.25 mm; BW: 2.75–2.95 mm; HW: 1.78–2.08 mm; PW: 1.88–2.13 mm. Biotopes: decaying, deciduous trees	***Hesperus* Fauvel, 1874**
–	Labrum not elongated (Fig. 14). Mandibles usually short, exceptionally only crossed in male pupa	***Bisnius* Stephens, 1829, *Gabrius* Stephens, 1829, *Philonthus* Stephens, 1829**

### Subtribe Quediina

**Table:** 

1	Abdominal spines and apical projections with sparse, tiny protuberances (Fig. 30). Body relatively slender. Antennae protruding slightly beyond apex of mid tibia. Hind tarsi reaching posterior margin of abdominal segments V (well visible III). BL: 5.5–6.0 mm; BW: 2.0–2.2 mm; HW: 1.5–1.6 mm. Biotopes: under bark of *Picea*, *Abies*, *Fagus* and *Acer*	***Quedionuchus* Sharp, 1884**
–	Abdominal spines and apical projections smooth, without protuberances (Fig. 31)	***Quedius* Stephens, 1829**

### Characters for phylogenetic analysis

1. Setiform projections or spines on the body (excluding segment IX): 0. absent, 1. present.

2. Protuberances on head: 0. absent, 1. present.

3. Number on protuberances on head: 0. 2, 1. 7.

4. Labrum, shape in outline: 0. U-shaped, 1. V-shaped.

5. Mandibles, shape of apices: 0. rounded, 1. pointed.

6. Maxillary palps, length: 0. protruding beyond apices of mid legs, 1. not protruding beyond apices of mid legs.

7. Antennae length: 0. at most reaching apices of mid tibiae, 1. protruding beyond apices of mid tibiae.

8. Protuberances on pronotum: 0. absent, 1. present.

9. Number of protuberances on pronotum: 0. about 10, 1. about 25.

10. Setiform projections on pronotum: 0. absent, 1. present.

11. Number of setiform projections on pronotum: 0. four, 1. more than four.

12. Length of setiform projections on pronotum: 0. shorter than half pronotum length, 1. at least half as long as pronotum.

13. Spines on pronotum: 0. absent, 1. present.

14. Protuberances on mesonotum: 0. absent, 1. present.

15. Pairs of tibiae with protuberances: 0. only mid, 1. mid and fore, 2. mid and hind, 3. all pairs.

16. Position of hind tarsi in relation to abdominal segments: 0. not adhering to abdomen, 1. adhering to abdomen.

17. Wing length: 0. at most reaching hind margin of segment III (I visible), 1. protruding beyond hind margin of segment III (I visible).

18. Structure of abdominal segments: 0. tergites and sternites fused into uniform rings, 1. tergites and sternites separate.

19. Abdomen shape: 0. gradually tapering from first to last segment, 1. tapering to last segment only in hind part.

20. Cuticular processes on sides of abdominal segment VII: 0. absent, 1. present.

21. Type of cuticular processes on sides of abdominal segment VII: 0. setiform projections, 1. spines.

22. Length of projections as spines on abdominal segment VII in relation to the width of that segment: 0. tiny, 1. long.

23. Cuticular processes on sides of abdominal segment VIII: 0. absent, 1. present.

24. Segment IX, terminal prolongations: 0. absent, 1. present.

25. Terminal prolongations, apical accessories: 0. absent, 1. present.

26. Apical accessories, shape: 0. straight, 1. curved.

27. Apical accessories, apex: 0. pointed, 1. rounded.

28. First pairs of spiracles, position: 0. in the same longitudinal line as others, 1. protruding laterally much more than others.

29. Appendages at functional spiracles of abdominal segments II–IV: 0. absent, 1. present.

### Phylogenetic analysis

**Figure 35.**
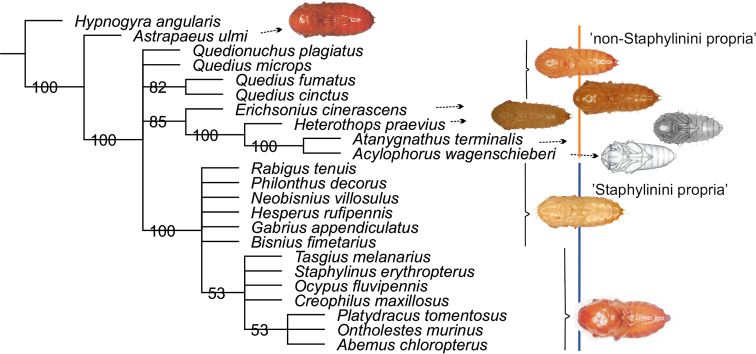
Fifty per cent majority rules consensus tree from a maximum parsimony analysis of 22 species of Staphylinini pupae.

The parsimony analysis retrieved 100 most parsimonious trees. The 50% majority rules consensus tree showed the following: i) separation of *Astrapaeus* from a clade of all other Staphylinini; ii) a well-supported clade of *Erichsonius*+*Heterothops*+*Atanygnathus*+ *Acylophorus*; iii) a well-supported clade of Staphylinini propria represented here by the subtribes Philonthina and Staphylinina (Fig. 35).

## Discussion

### Pupal morphology as an adaptation to environment

The occurrence of obtect pupae in the subfamily Staphylininae (including the tribe Staphylinini) is exceptional compared to other rove beetles and the majority of Coleoptera. The obtect pupa type with a compact body and usually heavily sclerotised cuticle appears to be far more resistant to negative impacts like attack from predators, parasitoids or mechanical damage. Therefore, an obtect pupa is probably a defensive adaptation. Presence of a tough cuticle in a pupa reduces the need for fully-grown larvae to construct a protective pupal cocoon, such as has been reported in a few Staphylinini species (*G. splendidulus* (Grav.), *O. murinus* (L.), *R. tenuis*) (Staniec 2004b; Pietrykowska-Tudruj and Staniec 2007; Staniec and Pietrykowska-Tudruj 2008c). Pupal cocoons are often encountered in representatives of the Aleocharinae, which have free, delicate pupae with weakly sclerotised cuticle (Frank and Thomas 1984; Staniec et al. 2010; Zagaja et al. 2014).

Another form of defensive adaptation is cuticular processes, which occur on various parts of the body (pronotum, abdominal segments) in almost all known pupae of the Staphylinini. They can take several forms: flexible, often arcuate setiform projections (e.g., *Gabrius*, *Philonthus*, *Staphylinus*), stiff spines (e.g., *Quedius*, *Erichsonius*, *Heterothops*) and, more rarely, convex protuberances (e.g., *Acylophorus*). These structures, besides having a defensive function (Hinton 1955), appear to minimise direct contact between the pupal body surface and the surrounding substrate (e.g., soil, leaf litter, plant remains, decaying wood), which probably allows the spiracles to function more efficiently. The number, length and shape of setiform projections or spines are also usually associated with the degree of cuticular sclerotisation and pupal body size. Their presence is particularly important for pupae with a weakly sclerotised cuticle, less resistant to damage. For this reason, such pupae usually bear numerous long, flexible, arcuate (especially on the pronotum) setiform projections (e.g., *G. splendidulus*, *N. villosulus* (Steph.) and most *Philonthus* species). In contrast, pupae with highly sclerotised cuticle bear far fewer such structures on the pronotum (e.g., *Quedius* sp. and *A. terminalis*), and in a few cases, the entire body surface is devoid of them (e.g., *A. wagenschieberi* and *A. ulmi*).

The number of setiform projections on the pronotum is a variable character within a species, the range of which is usually small (e.g., *R. tenuis* – 10–12, *P. nigrita* (Grav.) – 9–13), but occasionally much larger (e.g., *P. rectangulus* – 13–21, *P. politus* (L.) – 15–22). On small pupae there are usually fewer setiform projections on the pronotum (e.g., *G. splendidulus* – 7–9, *N. villosulus* – 7–8) than on much larger ones (*S. erythropterus* – 19–25, *P. decorus* (Grav.) – 22–24), a fairly obvious observation.

The pupae of some species from very wet habitats (*Atanygnathus terminalis*, *Acylophorus wagenschieberi*) exhibit special adaptations to their environment in the structure and localisation of spiracles (Staniec 2005a, b). They pupate among unsubmerged peat mosses (*Sphagnum*). However, water levels frequently vary in the peat-bogs they inhabit. The first pair of spiracles is thus especially large and protrudes strongly from the body outline, which facilitates gas exchange even at high levels of moisture. In the case of *A. terminalis*, the next three active spiracles additionally bear peculiar phylliform lobes. These probably serve to accumulate a supply of air in case the peat mosses are suddenly flooded or act as an additional respiratory surface (Staniec 2005b).

### Some comments on pupation

In the natural environment, larvae and adults of Staphylinini mostly live in the same microhabitats; pupation usually takes place there, too (Table 3). Only in the case of the above-mentioned species from wet microhabitats subject to flooding were pupae found in distinctly drier locations than those inhabited by mobile adults or larvae. These were usually unsubmerged layers of peat mosses, directly adjacent to higher-lying areas of bogs, 2–3 m from small bodies of standing water (Staniec 2005a, b). In the field, pupae of Staphylinini were found from spring to autumn (IV–X), although the pupae of most *Quedius* species were found in spring (IV–V). Among *Quedius*, only species confined to special microhabitats, such as tree hollows, crevices under tree bark or the vicinity of hornets’ nests, pupated during the summer (VII) or autumn (X). These phenological observations are in broad agreement with laboratory breeding data. In the laboratory, however, specimens from the same species pupated earlier (mainly V–VI) than in nature (Table 3), probably because of the more stable and warmer conditions (T = 20–24 °C) there, where the pupal stage lasted from 7 to 13 days, depending on the species. Larger species, e.g., *A. ulmi* and *S. erythropterus*, took distinctly longer to pupate than smaller ones, e.g., *R. tenuis*, *P. nigrita* (Table 3) (Staniec and Pietrykowska-Tudruj 2008a, c; Pietrykowska-Tudruj and Staniec 2012; Pietrykowska-Tudruj et al. 2014b).

**Table 3.** Data relating to known pupal stages of Staphylinini obtained from field and laboratory observations (Strassen 1957; Szujecki 1960, 1965; Staniec 1996, 1999a, b, 2001a, 2002, 2003a, b, 2004a, b, 2005a, b; Staniec and Kitowski 2004; Staniec and Pietrykowska 2005a, b; Pietrykowska-Tudruj and Staniec 2006b, c, 2007, 2010, 2011, 2012; Staniec and Pietrykowska-Tudruj 2007, 2008a, b, c; Staniec et al. 2009; Pietrykowska-Tudruj et al. 2014a, b; Vorst and Heijerman 2015).

Pupal stages observed	
In natural conditions	
Month/species	Microhabitat
IV/*Q. cinctus*, V/*Q. mesomelinus*, IV–V/*Q. cruentus*, VII/*P. varians*, VIII/*O. murinus*, *P. succicola*, *P. rectangulus*, *P. albipes*	rotting plant remains
V/*Q. fuliginosus*, *Q. fumatus*, *Q. humeralis*	moist leaf litter in woodland
VI/*P. corvinus*	rotting remains of *Carex* sp.
VI–VII/*A. wagenschieberi*, VI–VIII/*A. terminali*s	base of unsubmerged layers of peat mosses
VII/*Q. brevicornis*	tree hollow, in rotting wood
VII/*Q. plagiatus*	under protruding bark on a fir trunk lying on the ground
VIII/*S. erythropterus*	soil surface, under moss
IX/*P. quisquiliarius*	sandy river bank
X/*Q. dilatatus*	substrate under a nest of *Vespa crabro*
In laboratory conditions	
month/species	
II/*Q. brevis*, III/*Q. microps*, IV/*P. politus*, *Q. cruentus*; V/*B. nitidulus*, *P. corvinus*, *E. cinerascens*; VI/*G. splendidulus*, *H. praevius*, *N. villosulus*, *P. fumarius*, *P. micans*, *P. tenuicornis*, *O. fulvipennis*, *T. melanarius*, V–VI/*A. ulmi*, *G. astutus*, *P. cognatus*, *P. nigrita*, *P. punctus*, *P. lepidus*, *R. tenuis*, *S. erythropterus*; V–VII/*G. osseticus*, *P. rubripennis*, VI–VII/*H. rufipennis*, VII/*P. umbratilis*	

**Table 4.** Character matrix. ? missing data, – inapplicable characters.

**Taxon**	**Character**																												
	1	2	3	4	5	6	7	8	9	1	1	1	1	1	1	1	1	1	1	2	2	2	2	2	2	2	2	2	2
										0	1	2	3	4	5	6	7	8	9	0	1	2	3	4	5	6	7	8	9
*Abemus chloropterus*	1	0	-	1	1	1	1	0	-	1	1	0	0	0	2	1	1	1	1	1	0	-	1	0	-	-	-	1	0
*Acylophorus wagenschieberi*	0	1	0	1	1	1	1	1	1	0	-	-	0	1	2	1	0	1	0	0	-	-	0	1	0	-	-	1	0
*Astrapaeus ulmi*	0	0	-	0	1	1	1	0	-	0	-	-	0	0	2	1	0	1	1	0	-	-	0	1	1	?	?	1	0
*Atanygnathus terminalis*	1	1	1	1	0	0	1	1	1	0	-	-	0	1	2	1	0	1	0	1	1	1	0	1	0	-	-	1	1
*Bisnius fimetarius*	1	0	-	1	1	1	1	0	-	1	1	1	0	0	3	1	0	1	1	1	0	-	1	1	1	0	1	1	0
*Creophilus maxillosus*	1	0	-	1	1	1	0	0	-	1	1	0	0	0	2	1	1	1	1	1	0	-	1	1	0	1	?	?	0
*Erichsonius cinerascens*	1	0	-	1	1	1	1	1	0	0	-	-	0	0	0	1	1	1	1	1	1	1	1	1	1	0	0	1	0
*Gabrius appendiculatus*	1	0	-	1	1	0	1	0	-	1	1	1	0	0	2	1	1	1	1	1	0	-	1	1	1	0	1	1	0
*Hesperus rufipennis*	1	0	-	1	1	1	1	0	-	1	1	1	0	0	3	1	0	1	1	1	0	-	1	1	1	0	0	1	0
*Heterothops praevius*	1	1	0	1	1	1	1	1	1	0	-	-	0	0	2	1	0	1	1	1	?	1	1	1	1	0	0	1	0
*Neobisnius villosulus*	1	0	-	1	1	1	1	0	-	1	1	1	0	0	3	1	0	1	1	1	0	-	1	1	1	0	1	1	0
*Ocypus fulvipennis*	1	0	-	1	1	1	1	0	-	1	1	0	0	0	3	1	-	1	1	1	0	-	1	1	1	1	1	1	0
*Ontholestes murinus*	1	0	-	1	1	1	0	0	-	1	1	0	0	0	3	1	0	1	1	1	0	-	1	0	-	-	-	1	0
*Philonthus decorus*	1	0	-	1	1	1	1	0	-	1	1	1	0	0	3	1	1	1	1	1	0	-	1	1	1	0	1	1	0
*Platydracus tomentosus*	1	0	-	1	1	1	0	0	-	1	1	0	0	0	2	1	1	1	1	1	0	-	1	0	-	-	-	1	0
*Quedionuchus plagiatus*	1	0	-	1	1	?	1	0	-	0	-	-	1	0	?	1	0	1	1	1	1	1	1	1	1	0	?	1	0
*Quedius cinctus*	1	0	-	1	1	1	1	0	-	0	-	-	1	0	2	1	1	1	1	1	1	1	1	1	1	0	0	1	0
*Quedius fumatus*	1	0	-	1	1	1	1	0	-	0	-	-	1	0	2	1	1	1	0	1	1	0	1	1	1	0	0	1	0
*Quedius microps*	1	0	-	1	1	1	1	0	-	0	-	-	0	0	2	1	1	1	1	1	1	1	1	1	1	0	0	1	0
*Rabigus tenuis*	1	0	-	1	1	1	1	0	-	1	1	1	0	0	3	1	1	1	1	1	0	-	1	1	1	0	1	1	0
*Staphylinus erythropterus*	1	0	-	1	1	1	1	0	-	1	1	0	0	0	0	1	0	1	1	1	0	-	1	1	1	1	1	1	0
*Tasgius melanarius*	1	0	-	1	1	1	1	0	-	1	1	0	0	0	2	1	1	1	1	1	0	-	1	1	1	1	1	1	0
*Hypnogyra angularis*	1	0	-	1	1	1	0	0	-	0	-	-	0	0	0	0	0	0	1	0	-	-	0	1	1	0	?	0	0

### Phylogenetic potential of pupal characters for testing hypotheses of relationships within Staphylinini

Two major morphological groups were clearly distinguishable among the pupae of Staphylinini: ‘Staphylinini propria’, represented here by the subtribes Philonthina and Staphylinina only and forming a well-supported clade in our analysis, and ‘non-Staphylinini propria’, represented by the genera *Acylophorus*, *Astrapaeus*, *Atanygnathus*, *Erichsonius*, *Heterothops* and *Quedius*, whose systematic affiliation at the subtribal level has in recent years been the topic of much debate and has undergone far-reaching changes (e.g., Solodovnikov and Schomann 2009; Chatzimanolis et al. 2010; Brunke et al. 2016).

The critical characters distinguishing the two groups are: i) the presence of setiform projections on the pronotum in all species of the Staphylinini propria group, but their absence in non-Staphylinini propria; ii) the type of cuticular process on abdominal segment VII – setiform projections in the former clade, but spines in the latter (except for *Acylophorus*, which has no cuticular processes). Among the non-Staphylinini propria, a clade was recovered with species whose pupae bear protuberances on the body (the genera: *Acylophorus*, *Atanygnathus*, *Heterothops* and *Erichsonius*). These protuberances may be situated on the head, pronotum and mesonotum (*Acylophorus*, *Atanygnathus*), only on the head and pronotum (*Heterothops*), or only on the pronotum (*Erichsonius*). Pupae of *Quedius* do not possess any protuberances.

Since subtribal classification within the non-Staphylinini propria has undergone substantial changes in recent years. We discuss below the phylogenetic potential of the external pupal structures of some taxa in the light of such taxonomic revolutions.

### Pupa of *Astrapaeus*

There are practically no cuticular structures on the pupa of *A. ulmi*. The cuticular surface is devoid of any visible processes or protuberances (not including those on the legs of all pupae of the Staphylinini and the tiny accessories on terminal prolongations), which makes this species unique among the known pupae of the Staphylinini. Phylogenetic research based on adult and larval morphology, including fossil taxa, suggests that the monotypic genus *Astrapaeus* is not related to the subtribe Quediina (its traditional placement) but is a member of a rather isolated and basal lineage within Staphylinini (Solodovnikov and Schomann 2009; Solodovnikov 2012; Brunke and Solodovnikov 2013; Solodovnikov et al. 2013; Pietrykowska-Tudruj et al. 2014b). Based on molecular and morphological evidence, *Astrapaeus* is now included in subtribe CyrtoquediinaBrunke et al. 2016. Within Cyrtoquediina, a subtribe comprising species with mostly isolated distributions in the Neotropical, Oriental or Palaearctic regions (e.g., *Bolitogyrus*, *Cyrtoquedius*, *Parisanopus*, *Sedolinus*), only *Astrapaeus* occurs in and is restricted to Europe. The pupal characters of *Astrapaeus* support the isolated position of *A. ulmi* (and potentially other Cyrtoquediina) within the tribe, and outside the Quediina sensu Brunke et al. 2016. However, given the lack of data on the pupae of other members of the subtribe Cyrtoquediina, it is it is difficult to tell which morphological features of *Astrapaeus* are representative of the subtribe versus just genus level.

### Pupa of *Erichsonius*

Within *Erichsonius*, a genus including more than 160 species distributed over almost all the world, the pupal stage is known for just three: the Nearctic *E. alumnus* Frank and *E. pusio* (Horn) (Schmidt 1996) and the Palearctic *E. cinerascens* (Grav.) (Pietrykowska-Tudruj and Staniec 2006c). Until the end of the 20^th^ century, this genus was placed the subtribe Philonthina. But many recent phylogenetic analyses of adults utilising morphological and molecular data have indicated that the original placement of *Erichsonius* was incorrect (e.g., Brunke and Solodovnikov 2013; Chani-Posse 2013; Brunke et al. 2016). Initially, the genus was withdrawn from Philonthina and allocated to the Anisolinina grade within Staphylinini propria. However, the latest analyses show that *Erichsonius* is monophyletic and forms a separate subtribe Erichsoniina (Brunke et al. 2016, Chani-Posse et al. 2018).

The pupa of *Erichsonius* possesses a series of characters clearly distinguishing it from species classified among Staphylinini propria. They are: i) a lack of setiform projections on the pronotum; ii) the presence of protuberances on the pronotum; iii) cuticular processes on the abdominal segments in the form of spines. At the same time, these characters are shared with species of four genera of non-Staphylinini propria, i.e., *Atanygnathus* and *Heterothops* (all characters), *Acylophorus* (characters i and ii) and *Quedius* (characters i and iii). The results of our analyses suggest *Erichsonius* is distinguished from all other non-Staphylinini propria with known pupae by the number of protuberances on the pronotum. Pupae of *Erichsonius* have few protuberances (10 at most), whereas they are more numerous (more than 10) on the pupae of other taxa. Since the pupal stage is unknown in many other genera of Staphylinini and *Erichsonius* species, it is hard to assess the extent to which the number of protuberances is consistent within and unique to the genus. Given the present state of knowledge of pupae, we can regard it as unique to *Erichsonius*, and therefore evidence in favour of the recently erected subtribe Erichsoniina (Brunke et al. 2016).

### Pupa of *Heterothops*

Within *Heterothops*, a globally distributed genus with 149 described species, the pupal stage is known only in *H. praevius* (Herman 2001; Pietrykowska-Tudruj and Staniec 2006c). This poorly defined genus was moved from the conventional subtribe Quediina and initially included in the large lineage Tanygnathinina sensu Solodovnikov and Schomann 2009; later it was placed in the subtribe Amblyopinina, containing fauna mainly from the Neotropical and Australian regions (Solodovnikov and Schomann 2009; Assing and Schülke 2012; Solodovnikov 2012).

Our analyses have demonstrated that the pupa of *Heterothops* has many characters in common with *Atanygnathus*. They are: processes on the head and pronotum, spines on abdominal segments II–VII, broad elytra, short hind leg tibiae (not reaching the lateral margin of the body), protuberances on the mid and hind legs, and long antennae. There are not many characters (not present in Staphylinini propria) shared between *Heterothops* (*H*) and *Quedius* (*Q*) (spines on abdominal segments and protuberances on the mid and hind tibiae), whereas there are many differences: head size (in proportion to the rest of the body) (small – *H*, large – *Q*), protuberances on the head and pronotum (present – *H*, absent – *Q*), antenna length (long – *H*, short – *Q*), width of elytra (wide – *H*, narrow – *Q*). In the light of current knowledge of Staphylinini pupae, one can state unequivocally that the morphology of *Heterothops* pupae supports the separation of this genus from the subtribe Quediina. There are several recent studies based on adult characters, or in combination with DNA that have confirmed its placement within the subtribe Amblyopinina (e.g., Brunke et al. 2016; Chani-Posse et al. 2018; Brunke et al. 2019). Among the 17 genera forming this group, only the pupa of *Heterothops*, the single taxon in this group which occurs beyond the southern hemisphere, is known (Brunke et al. 2016).

The present study has shown that the external structures of Staphylinini pupae could be a useful, alternative source of evidence for resolving the relationships of some higher taxa within the tribe. However, much more descriptive work is needed – mainly expanding the data matrix to include new species/genera and compiling new morphological data. Unfortunately, the pupae of many species of phylogenetic interest will probably remain unknown owing to the great difficulties with their collection and identification.
